# Secondary use under the European Health Data Space: setting the scene and towards a research agenda on privacy-enhancing technologies

**DOI:** 10.3389/fdgth.2025.1602101

**Published:** 2025-06-19

**Authors:** Sarah van Drumpt, Kartik Chawla, Tom Barbereau, Dayana Spagnuelo, Linda van de Burgwal

**Affiliations:** ^1^ Netherlands Institute for Applied Scientific Research (TNO), The Hague, Netherlands; ^2^ Vrije Universiteit Amsterdam, Amsterdam, Netherlands

**Keywords:** European Health Data Space, data permit, ethical secondary use, digital health, privacy enhancing technologies

## Abstract

The Regulation for European Health Data Space (EHDS) aims to address the fragmented health data landscape across Europe by promoting ethical and responsible reuse of data, seeking to balance the opportunities for data reuse with the risks it entails. However, the techno-legal aspects of navigating this balance remain poorly understood. This study adopts a qualitative and inductive approach, using semi-structured interviews to explore the risks, challenges, and gaps in the implementation of privacy-enhancing technologies (PETs) within EHDS, particularly in the context of its governance structure and data permits for secondary data use. The findings identify five distinct categories of concerns, based on fourteen risks, and highlight seven governance and technological solutions, illustrating how these solutions address multiple, often correlated risks. The interdependence between concerns and solutions emphasises the need for a strategic and integrated approach to both governance and technology. This mapping between the risks and solutions also highlights the central role of certain solutions, such as public engagement and awareness, in addressing multiple risks. Furthermore, it introduces a new dimension to the concerns by focusing on the structural imbalances in access to the health data economy. We conclude by proposing a research agenda to advance the integration of PETs into the EHDS framework, ensuring that data permits can effectively facilitate secure, ethical, and innovative health data use.

## 1 Introduction

The landscape of European health data is fragmented. To date, organisations, researchers, and the government have found it increasingly difficult to leverage existing data, which are often not findable, accessible, interoperable, or reusable (1, 2). In response and to strengthen the resilience of the healthcare system, the European Commission’s data strategy is currently establishing the European Health Data Space (EHDS) (3). This initiative comprises of rules, standards, and practices aimed at stimulating cross-organizational collaboration and secondary use of data in healthcare, all while in adherence to the General Data Protection Regulation (GDPR) (4).

The EHDS was established into law through Regulation (EU) 2025/327, officially entering into force on March 26, 2025. This regulation evolved from and significantly expands upon the existing Cross-Border Healthcare Directive (2011/24/EU), which had initially set the groundwork by enabling EU Member States to voluntarily exchange digital health data, particularly ePrescriptions and Patient Summaries, through the MyHealth@EU platform. The EHDS formalises and broadens the digital sharing of health information, including this cross-border exchange of data. The EHDS regulation applies from 26 March 2026, though different aspects thereof shall apply in a staggered manner as described in Art. 105.

The EHDS holds significant promise to advance healthcare by improving data accessibility for research, innovation, and policy making (5). However, several challenges remain. Privacy and security concerns, for example, are central, as the sensitive nature of health data requires robust safeguards to protect individual rights (6). The complexity of ensuring informed consent (or, in the case of the EHDS, ensuring that the patients are appropriately informed in order to opt-out if they wish to do so) in large-scale secondary use of data initiatives also raises ethical issues, particularly in ensuring that individuals fully understand how their data will be used. Furthermore, ensuring equitable access to health data and preventing potential misuse remains a critical challenge, especially in balancing the benefits of secondary use with respect to individual autonomy and confidentiality (7). Tensions can also arise between different values, such as patient privacy and the value of new treatment protocols (5, 8–10).

Creating conditions for ethical secondary use of data involves a threading the needle between all of these varied concerns, and maintaining the appropriate equilibrium between regulatory compliance and technological security. These conditions are set in the overall governance structure of the EHDS and, crucially, in data permits. Data permits are a critical prerequisite for collaboration, and are required to be submitted by Data Users (e.g., insurance companies, researchers) to a Health Data Access Body (HDAB), which must correspondingly determine whether to allow access to health data stored by Data Holders (e.g., health institutions collecting patients’ data) for secondary use or not. However, while some researchers have pointed towards Privacy-Enhancing Technologies (PETs) as promising solutions to address some of the concerns in European data spaces (11, 12), little is known about how to approach this careful balance from a techno-legal perspective.

In response, this research considers three interconnected research questions:

RQ1: What are the risks associated with the EHDS for secondary use of health data?
RQ2: What are potential solutions to address these problems?
RQ3: What are research gaps in the adoption of PETs in the EHDS?

To answer these questions, the paper is structured as follows. Section 2 considers the background on the EHDS and PETs. Section 3 introduces the qualitative, inductive research method followed. Section 4 presents the findings from the qualitative data collection and analysis; notably, (4.1) a systematic overview of risks associated to the EHDS categorised in terms of concerns (RQ1), (4.2) solutions to address these risks (RQ2), and (4.3) exploring the gaps for applying PET (RQ3). Section 5 discusses these findings and highlights a research agenda for PETs in the EHDS. We conclude in Section 6.

## 2 Background

### 2.1 Primer on the European Health Data Space

Proposed in 2022, the European Health Data Space (EHDS) is a flagship initiative of the European Union designed to transform the secondary use and sharing of health data (3). The creation of the EHDS – among other Common European Data Spaces in domains such as agriculture or manufacturing (13) – is a central part of the European strategy on digital data. The purpose of each data space is to establish common data infrastructures and governance frameworks, which facilitate data pooling, access and sharing (14).

Two of the primary goals of the EHDS, in line with the GDPR, are to facilitate natural persons’ access to and control over their personal electronic health data, in the context of healthcare, as well as facilitating achieving “other purposes that involve the use of electronic health data in the health and care sectors” (EHDS, Recital 1). By facilitating secondary use of health data, the EHDS aims to boost research, innovation, and policy making (EHDS, Art. 53). Through the EHDS, researchers and policy makers can have access to *anonymised*^1^ data sets to drive advances in medical treatments, improve public health strategies, and develop data-driven healthcare policies.

One of the cornerstones of the EHDS is the creation of a “legal obligation on data holders to share electronic health data for secondary purposes if certain conditions are met” (10). Under the EHDS (3), any individual or organisation can request access to electronic health data (“data user”) from a data holder (“any natural or legal person, which is an entity or a body in the health or care sector, or performing research in relation to these sectors”) through a data request, as long as they meet the requirements set out in Chapter IV. Despite the legal obligation to share data for secondary use, access may be subject to fees to cover costs related to assessing a data request, preparing and making data available (EHDS, Art. 62). This refers to data collected primarily through clinical care or research, and encompasses electronic health records, socioeconomic, environmental and behavioural data, healthcare-related administrative data, genetic information, among others (EHDS, Art. 51). Data users can reuse this data for secondary purposes (EHDS, Art. 53(1)), including scientific research, algorithmic training, and personalised care through the Health Data Access Bodies (EHDS, Art. 55). Within the scope of this paper, we focus on secondary use of health data, and particularly in the context of the second method of access, where multiple players are involved share responsibilities over the data processing and demonstration of compliance with regulations, and where tensions or uncertainty might arise.

**Figure 1:**
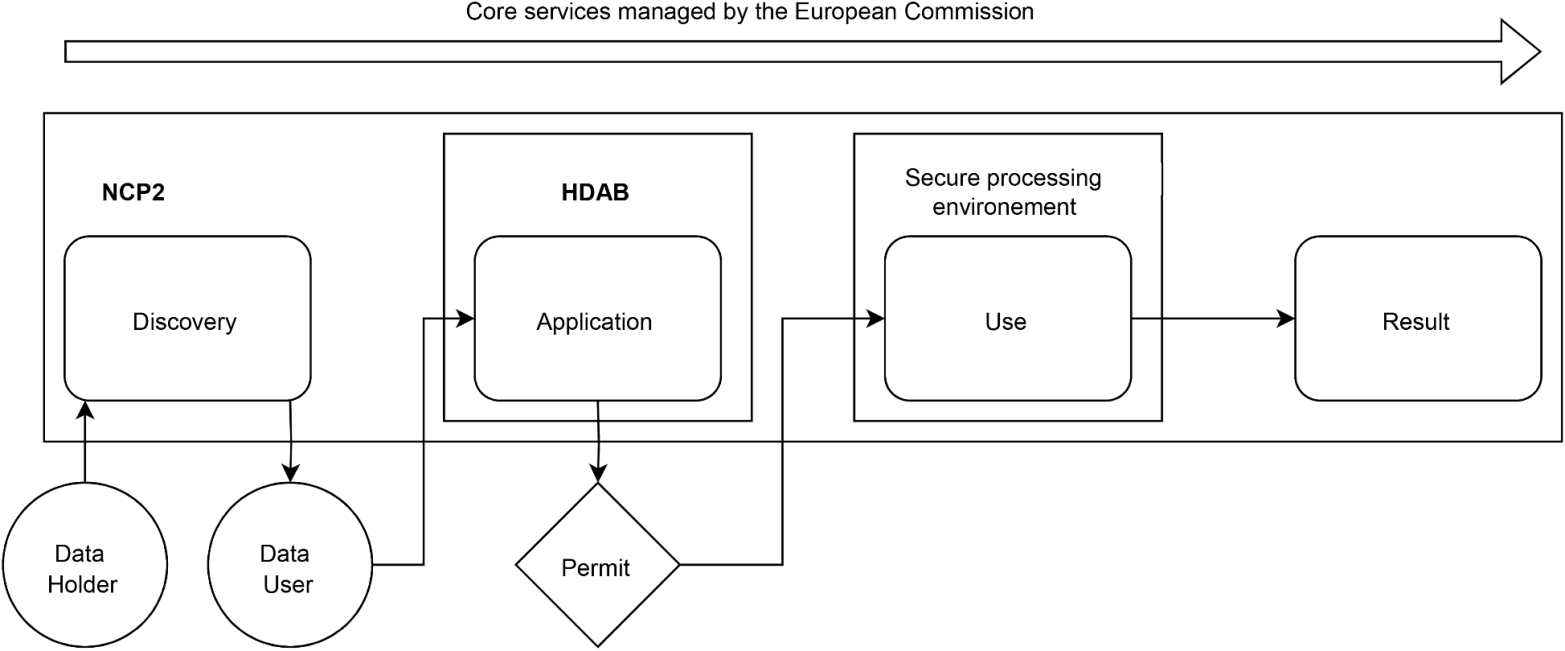
Process for managing secondary data use by a national contact point for the secondary use of health data. NPC2: National contact point for secondary use of health data; HDAB: Health Data Access Body.

In Figure 1, based on Kalliola et al. (12), we sketch out the process around the secondary use of data as managed by a national contact point for the secondary use of health data (NCP2). There are four main steps related to the types of data interactions: (1) discovery; (2) application (to a permit); (3) actual secondary use (in a secure processing environment); and (4) publication of results. The entire process is supported by core services managed by the European Commission.

Overall, the EHDS — while ambitious (5) — represents a transformative step towards a more interconnected, efficient, and patient-centred healthcare system across Europe. It has the potential to enhance individual healthcare experiences, stimulate innovation, and improve health outcomes throughout the continent. However, as mentioned, it also raises significant concerns, particularly regarding its implications for research, legal frameworks and broader societal impacts (5, 8). Experts in health policy, law, ethics and social sciences have cautioned that the EHDS could undermine patient control over data, impede healthcare and research initiatives, and diminish the public value of health data sharing—issues that requires substantial policy adjustments to realise the system’s intended benefits (5). These are the risks that we explore further in depth in this paper.

### 2.2 Privacy-enhancing technologies in health

In an era where personalised medicine and large-scale data sharing are transforming healthcare, the need for strong privacy protections has grown. The need for these protections - coming from both legal as well as ethical requirements - clashes with the ever increasing need for access to greater amounts of data. The term Privacy-Enhancing Technologies (PETs) is an umbrella term that refers to a set of innovative methods that address this (15, 16), by allowing sensitive data to be shared, analysed, and/or used without undesirable exposing sensitive details (in health, individual patient information for example). These technologies, such as federated learning, secure multi-party computation, and so on, help balance the need for valuable medical insights with the demands for strong privacy assurance. Different PETs offer different benefits and trade-offs, making them suitable for various healthcare applications (11). For example, some methods prioritize data utility, while others offer stronger privacy guarantees at the cost of increased computational complexity or reduced model performance. Some focus on input privacy (e.g., federated learning) and enable linking data across sources to a single individual (e.g., secure multi-party computation), others target output privacy (e.g., differential privacy), while some allow verification without disclosing the underlying data (e.g., zero-knowledge proofs). To further explore the scope and implementation of PETs, readers may refer to the detailed guidance offered by the Information Commissioner’s Office (17).

Their potential is already visible in several real-world use cases. For example, federated learning in medical imaging enables hospitals and research institutions to train artificial intelligence (AI) models on images without sharing raw patient data (18, 19). This improves diagnostic accuracy while ensuring that patient records remain secure. In Secure Genomic Data Analysis, advanced encryption methods, such as homomorphic encryption – which allows data to be analysed while still encrypted (20) – and secure multiparty computation – which enables multiple parties to work together on a data without revealing their individual inputs (21) – allow researchers to study genetic factors in diseases without exposing sensitive genetic information.

Despite these advances, PETs still face significant challenges before they can be widely adopted in healthcare. Some methods require significant computing power, making them slow for real-time applications (22). Others face technical hurdles, such as ensuring different healthcare systems can work together, meeting strict legal requirements, and developing clear ways to measure how well these privacy protections work (23). Overcoming these challenges will require collaboration between medical professionals, data scientists, legal experts, and policymakers (16).

Integrating PETs into data spaces presents both an opportunity and a challenge. Within healthcare, data spaces could facilitate secure and privacy-preserving access to diverse health datasets, enhancing research, innovation, and patient care. However, as highlighted by the Towards European Health Data Spaces project and recent studies, the integration of PETs into these infrastructures remains largely theoretical (11, 12, 15). While PETs provide mechanisms to safeguard privacy, their practical deployment within data spaces is hindered by technological, legal, and governance complexities. The absence of proven frameworks that effectively combine PETs with data space architectures reflects a gap in both research and implementation. Bridging these gaps will require interdisciplinary collaboration to establish standardised, scalable, and interoperable approaches that can ensure both data protection and utility within emerging data spaces (15, 24).

## 3 Method

This research followed an inductive, empirical approach whereby semi-structured interviews are the primary source of data (25) and qualitative coding techniques the method of analysis (26, 27). This approach was selected given the relative lack of available empirical knowledge on the EHDS, and on the application of PETs in that context.

### 3.1 Data collection

For the study, we carried out 16 semi-structured interviews with experts in the broader field of healthcare whose work relates to the EHDS. The interviews were conducted as part of a research project funded by the Netherlands Organisation for Applied Scientific Research (TNO) over the course of three months (November 2024–February 2025). Interviewees were selected on the basis of their topical expertise as well as their affiliation (Supplementary Material). The core condition for the selection of experts was their knowledge of the EHDS, their involvement in preparatory efforts, policy discussions, data governance structures, research or implementation planning related to EHDS processes. The group of experts is interdisciplinary by nature and it considers both, public and private sector affiliations. They are understood as agents with implicit and relevant knowledge on the topic of investigation (25).

Semi-structured interviews were selected as they are the most appropriate in cases were little knowledge is available given their ability to produce rich insights from a local, case specific context (25). The semi-structured interviews were conducted following an interview guide (Supplementary Material) that served to streamline coverage of two principal thematic areas (28): data permits and secondary use of health data. For each interview, the questions were slightly adapted to match the interviewee’s position, background and expertise (29). The interviews were held via video conference and lasted around 60 min on average. Each interview was recorded and subsequently transcribed and coded.

### 3.2 Data analysis

In consideration of the research questions, and to identify patterns in the qualitative data, we followed the conventional two stage coding process (26, 27). To code the data, we used the qualitative data analysis software ATLAS.ti. Additionally, we employed its Conversational AI function to validate the categories and assess the completeness of supporting quotes, using anonymised interview transcripts to mitigate the risk of re-identification of interviewees. All interviews were conducted in the presence of two researchers to enhance reliability and reduce interviewer bias. The initial coding book was developed by one researcher and tested on a sample basis by the other interviewers to ensure consistency and clarity. A fourth researcher, who was not involved in the data collection, independently reviewed a subset of the coded material to provide external validation. The research team represented diverse disciplinary backgrounds, including public health, information technology, law, and governance, which supported reflexivity and enriched the analysis. Table 1 provides samples of how we coded statements.

**Table 1:** Coding stages of the qualitative data analysis.

Statement	1. Open Code (*risk*)	2. Selective Code (*concern*)
The confidentiality […] is lost because neither the patient nor the doctor can guarantee that that confidentiality will exist in the future (I10)	Breakdown of patient-doctor confidentiality	Patient-doctor relationship
A vulnerability of the whole EHDS scheme again, is the extent to which value created with the research project will trickle down to society (I15)	Lack of public benefit from data reuse	Public benefit vs. commercial gains
Privacy would need to come first in a way. […] If you ever have privacy breaches or patient data being accessed in an unauthorized way, then this would be a huge scandal of course. (I4)	Privacy breaches	Data security

*Stage 1. Open coding:* During the first stage, open coding, we assigned initial codes that captured key ideas or actions and loosely grouped them in terms of *risks*. These codes were descriptive and closely aligned with the language of the participants, ensuring that our analysis remained grounded in their perspectives (26). As group of researchers, by engaging in an iterative and dynamic process of debate, we refined and expanded the codes as new insights emerged during repeated engagement with the data (30). This stage allowed us to explore the data in an open and unbiased manner, laying a solid foundation for the subsequent stages of analysis by organizing it into manageable and meaningful segments.

*Stage 2. Selective coding:* We built on the initial open coding by identifying relationships and connections between the codes to form broader categories and themes in terms of *concerns*. This stage involved systematically organizing the data around central concepts, focusing on how different codes interacted within specific contexts, causal relationships, and outcomes (26). This process enabled us to move beyond descriptive analysis, allowing us to develop a deeper understanding of the data and to answer our research questions. Throughout both stages we also identified a number of ways to address the risks, labelled as solutions. We identified these in Stage 1 and grouped them under one, separate category from the concerns in Stage 2.

## 4 Results

### 4.1 Concerns and risks

We present the results in terms of risks which we mapped to a higher-order category of concerns. Table 2 provides an overview of these. In the subsequent sections, we discuss each of the identified risks.

**Table 2:** Concerns and associated risks.

Category (concerns)	Risks
Patient-doctor relationship	Loss of confidentiality
	Withholding information
	Avoidance of healthcare services
Public benefit and commercial gains	Commercial exploitation
	Lack of public benefit from secondary data use
	Unequal access to the health data economy
Regulation vs. innovative capacity	Over-regulation as a barrier to innovation and oversight
	Ambiguity in roles, responsibilities, and enforcement
Control and consent	Coarse opt-outs due to lack of granularity
	Trust and transparency
	Ensuring informed consent
Data accuracy and minimisation	Loss of context and misinterpretation
	Lack of incentives for quality assurance
	Enforcing data minimisation

#### 4.1.1 Concern: patient-doctor relationship

The interviewees mention concerns that secondary use of health data will become detrimental to the traditional patient-doctor relationship.

##### 4.1.1.1 Loss of confidentiality

Interviewees raised concerns about the potential impact that implementation of the EHDS may have on the doctor-patient relationship, particularly the potential detriment of “confidentiality” (I4; I7; I8; I10; I11) and “trust” (I10; I15). The fear that “the confidentiality between patient and doctor is lost because neither the patient nor the doctor can guarantee that confidentiality will exist in the future” (I10) underscores this risk.

##### 4.1.1.2 Withholding information

Patients may withhold information due to uncertainty about data privacy, leading to a shift in the traditional dynamics of medical consultations. As one interviewee noted, “they fear that, well, they will not be able to be sincere, to tell everything” (I7). Additionally, the perception that “commercial access to health data” threatens trust between patients and healthcare providers was emphasised, with concerns that “people trust in it [the doctor-patient relationship], and it is a larger risk than people account for” (I15). This fear of secondary use of data may, in turn, discourage patients from fully disclosing information during medical visits, potentially compromising the recieved care.

##### 4.1.1.3 Avoidance of healthcare services

Interviewees pointed out that consequence of this distrust is the potential avoidance of healthcare services (I7; I10). One interviewee expressed that “if it goes like this, then I will just not go to the doctor, so they will avoid care” (I7), suggesting that diminished trust in medical confidentiality may lead to long-term public health challenges. If patients fear that their health data may be repurposed beyond their control, they may be reluctant to seek medical attention, undermining (preventive) care efforts.

#### 4.1.2 Concern: public benefit and commercial gains

The interviewees raised the concern that health data within the EHDS may be used disproportionately for private commercial profit rather than public benefit.

##### 4.1.2.1 Commercial exploitation

The potential for “commercial and private entities” to access sensitive data was seen as controversial (I3; I4; I7; I8; I10), with fears that individuals would “pay twice, first with [their] data for big companies, and then again for expensive medicines” (I7). This perception of economic exploitation raises ethical concerns about whether large corporations will return the value derived from health data “to society and not to the shareholders’ pockets” (I7). Another interviewee expressed the need to “not make some pharmaceutical companies more rich [*sic*] to say very bluntly” (I16). There is also a mention of the potential risk that “hospitals are going to see the data that they acquired from patients as something that they can sell and make money on” (I11).

##### 4.1.2.2 Lack of public benefit from secondary data use

Interviewees pointed out that health data is “a profitable product” (I4; I16), and questioned “how can these gains feed back into the community?” (I4) and wondered how to make this secondary data use “for the public good, [to] make our lives better” (I16). The lack of mechanisms to ensure that “value created with research projects will trickle down to society” (I15) was highlighted as a structural vulnerability of EHDS.

Additionally, public perception plays a crucial role. If citizens believe their “sensitive personal data is out there for not the right purposes,” they are more likely to “enforce their right to opt out” (I16). This, in turn, creates “a really large risk that a lot of opt-outs will come” (I15), ultimately undermining the system.

Furthermore, interviewees stressed that public benefit cannot be realised if data use exposes individuals to harm (I8; I14). Beyond concerns about value distribution, they pointed to ethical risks such as misuse by commercial actors. One interviewee noted the challenge of determining who should access data, warning of “a risk of maybe misusing data, like insurance companies and so on” (I8). Safeguards against such misuse are therefore important to ensure that public benefit is both meaningful and ethically sound.

##### 4.1.2.3 Unequal access to the health data economy

A recurring risk raised by the interviewees was that EHDS may disproportionately benefit large corporations while excluding smaller organisations. The complexity and resource requirements of obtaining data access were seen as a potential barrier that may prevent smaller organisations from having the same access as larger ones. Interviewees highlighted that navigating the EHDS application process requires significant expertise, time, and resources, which are more likely to be available to larger organisations. As one interviewee noted, “only the very big companies can do these kinds of things because they have the deep pockets” (I11). There were calls to “ensure that it’s not just the big companies who benefit from this most [*sic*]” (I4), and to prevent a scenario where standardisation leads to entities with a lot of resources becoming dominant players and monopolising research opportunities (I13). Without proper safeguards, “small and medium enterprises may struggle to access data” (I16), reinforcing existing inequalities in the health data economy. These concerns reflect fears that, despite the goal of the EHDS to make data more accessible, the process may inadvertently favour those who already have the capacity to meet its demands.

#### 4.1.3 Concern: regulation vs. innovative capacity

The interviewees raised the concern that excessive regulation within the EHDS could hinder innovation and the capacity for progress in the healthcare sector.

##### 4.1.3.1 Over-regulation as barrier to innovation and oversight

Interviewees raised the risk that the introduction of yet another regulation could further complicate an already complex legislative landscape, hindering collaboration and slowing innovation, particularly for smaller companies. Fragmentation was seen as “hampering innovation and speed of innovation, certainly by small companies, because you need time and deep pockets to really get there” (I11). Differences in national regulations add to this challenge, as “some countries already had some national regulations about secondary users” (I8), making alignment across borders difficult.

Furthermore, overlapping regulatory frameworks – such as GDPR, Medical Device Regulation (MDR), and the AI Act –, were seen as creating confusion. One interviewee noted that “there is a lot of cross-stop between different regulations at the moment” (I16). Beyond the impact on innovation, excessive regulatory complexity also threatens public trust. If procedures are perceived as too intricate or opaque, individuals may disengage from the system altogether. As one interviewee warned, “probably the worst thing that can happen to us is to lose trust. And the reason is that the procedures are complex” (I7). Without clear, harmonised guidelines, both industry and citizens may struggle to navigate the system, ultimately undermining its intended benefits.

##### 4.1.3.2 Ambiguity in roles, responsibilities and enforcement

Another risk raised by interviewees is the ambiguity surrounding roles and responsibilities in health data governance. While many referred to “owner” or “ownership” (I3; I6; I11; I14) of data, this term is not explicitly defined within the EU regulatory framework. Instead, their concerns appeared to relate to the rights and obligations associated with data use and control. As one interviewee questioned, “if you combine from two different sources […] then who’s [responsible] for the new data set? And that is something which has not been defined yet” (I14).

In addition, the mechanisms of “enforcement” (I14, I16) are also uncertain. Interviewees expressed concerns about accountability, asking, “who checks these things right? Who checks the archiving? Who checks the obligation to publish the results? Who checks the information to the data holder?” (I16).

#### 4.1.4 Concern: consent and control

##### 4.1.4.1 Coarse opt-out due to lack of granularity

Interviewees raised the risk that broad, non-specific opt-out options could undermine the benefits of data sharing. One interviewee warned about “the opt-out system that is not granular and people might opt out a lot […] [Subsequently] it won’t have the benefit that we want” (I15). The lack of granularity in the opt-out system would pose issues, as “the opt-out is a bit an all or nothing […] if this is just about sound research by public organizations, then you might not have a problem with it. Well, you would have a problem with it when a commercial party would take that data to improve systems or improve medical technologies” (I8). Furthermore, interviewees highlighted that people are generally reluctant to share data freely: “people really don’t want […their] data to be available just like that” (I7).

Lastly, the lack of options for differentiating between types of data usage was also mentioned. It was noted that “if you cannot differentiate […] maybe I will say ‘okay’, the thing of mental health weighs heavier than the thing on my thumb, so I will say ‘no’ to all” (I12). This is an indication for a need for more nuanced consent options.

##### 4.1.4.2 Transparency and trust

Interviewees agreed that ensuring transparency was essential for maintaining trust, but also noted that failure to communicate effectively could lead to widespread scepticism. One participant noted, “if people do not have the opportunity to know what is happening […] I am afraid that there are going to be certain sectors of society that will just catalyse a complete anti-movement” (I9). Furthermore, interviewee 9 pointed out that vulnerable groups might already have low trust in the system. Another interviewee remarked that “once you lose trust […] people would just stop trusting the system […] when you’re talking about vulnerable groups, the trust is already low in the system” (I7).

In this context, some interviewees emphasized that transparency should foster trust, but that if data usage was perceived as vague or not beneficial to the public, it could lead to distrust. One participant cautioned that “this transparency should enhance trust. But if there’s all the time published [*sic*] that data is used for use cases where the public doesn’t necessarily understand the benefit of yet […] it can be interpreted as something that is not beneficial” (I13).

##### 4.1.4.3 Ensuring informed consent

Interviewees raised concerns regarding the challenges of obtaining informed consent under EHDS, particularly in relation to the complexity of explaining how health data is reused and the difficulties in ensuring patient comprehension. It was emphasised that the informed consent process would be challenging due to the uncertainty surrounding the use of health data. One participant noted: “the informed consent procedure will become rather challenging because a patient does not know what’s going to happen with their data” (I3). It was widely supported that the complexity of the consent process might lead to confusion, with interviewees agreeing that explaining this to all patients would be difficult (I3; I7; I9). As one participant stated, “it’s really naive to think that we will be able to explain this to everyone” (I7), highlighting the risk that many patients would struggle to fully understand how their data would be used. Health practitioners are not suspected to have the time to explain how the data registration might be repurposed for secondary use, as stated by another interviewee, “I don’t think we can expect that they have time to explain this to the [patients]” (I9). The issue of information overload was also raised, with interviewees stressing the need for a more structured approach to presenting information (I5; I7). One interviewee suggested: “please make it layered […] so people always ask me how to inform patients. They don’t understand” (I7), underscoring the importance of a tiered system that would allow patients to better process and retain the necessary information about data usage.

#### 4.1.5 Concern: data accuracy and minimisation

##### 4.1.5.1 Loss of context and misinterpretation

Some of the interviewees raised concerns that the data made available through the EHDS may not be as useful as hoped for. This is expected due to the loss of contextual information that makes it useful. One interviewee noted that “you lose information about the context if you are not the data holder, hence more prone to wrong interpretation. Therefore knowledge transfer should be done right” (I4). The separation between the data holder and data user may make this worse as “the quality of research is improved through contact with the data holder. And that link has severed under the EHDS scheme which is a very apparent vulnerability in the new system,” adding that it is a problem that is “overlooked” (I15).

Furthermore, data entry practices may be different across medical facilities and even departments within the same facility, and “as a result, when datasets are merged, inconsistencies arise due to varying data definitions.” (I3) The measures used may also vary similarly: “health data is messy. It’s just very messy data. And everywhere, even per hospital, you can have seven different ways in which like a flu virus is being measured” (I4).

When data is used outside its original context, researchers may misinterpret its meaning. “When you do your research and draw conclusions from the data, you present it to the database holders […] and then they go like, you can’t interpret that from our data. It’s not meant that way” (I15). Cross-border data sharing intensifies this issue, as “if somebody from Italy comes to the Netherlands and asks for the data, there’s always the risk of misinterpretation because they don’t know why or how the data was originally collected” (I8).

##### 4.1.5.2 Lack of incentives for quality assurance

Interviewees raised the concern that there may not be sufficient incentives to ensure appropriate data quality, as improved data quality does not create any benefits for the individuals entering the data in the system. A separation can further be made here between the incentives for data holders, illustrated by “[…] [data holders] can only charge for the time spent preparing the data, not for maintaining the database itself. This creates financial sustainability issues for research databases, impacting researchers, patients, and healthcare providers alike” (I15) and clinicians or doctors entering the data. In other words, “the way that this data is registered doesn’t follow these quality standards, because clinicians or the doctors introducing this data in their clinical setting […] follow their own ways of stating things. The quality is not their main aim” (I2).

##### 4.1.5.3 Enforcing data minimisation

One interviewee raised concerns regarding the lack of available clarity on the metadata requirements. The interviewee stated, “we have raised multiple times, the current metadata format limits the way you can express the data minimisation […]. It does not have the capability to state variables, to define the variables that are in a given dataset. It just describes the dataset. So you can request a full dataset, which doesn’t follow the minimisation concept.” (I2).

### 4.2 Solutions

Two higher order categories of solutions emerge from the data. First, we grouped those solutions together which pertain to governance and organisational processes (policy). Second, we grouped those solutions that pertain to technological tools. Table 3 provides an overview of these. In the subsequent sections, we discuss each of the identified solutions.

**Table 3:** Overview of solutions.

Category	Solution
Governance & Policy	Data governance & responsibility
	Standardization & compliance
	Public engagement & awareness
	Consent & opt-out Mechanisms
Technological	Access control & data minimisation
	Federated Systems & Privacy-enhancing technologies
	Data quality assurance

#### 4.2.1 Governance & policy

##### 4.2.1.1 Data governance & responsibility

Interviewees proposed solutions that we broadly categorised under improvements to data governance, responsibilities, and accountability. Specifically, “part ownership and responsibilities” need to be better defined and “mandated” because “if you have the clear rules and responsibilities and ownership, then someone can also be accountable for it” (I14). In terms of data management, it suggested that data be kept “at the sensor itself,” to “keep it as much as possible at its source,” (I2) advocating for the maintenance of data control at its origin.

Adding to these points, Interviewee 4 emphasised that the concept of public value should be carefully defined together with the “value that is actually added.” That definition, should be developed “in consultation, with communities who actually face specific diseases or health conditions” (I4). Similarly, it was highlighted that the importance of a clear definition of public interest, noting that “you have to have a clear definition of public interest coming from national law or policy somewhere. […] It would be very good if this definition of public interest is at the EU level, because what you will get is that people are going to shop data.” A unified definition at the EU level could mitigate the issue of data “shopping” across different jurisdictions (I7).

##### 4.2.1.2 Standardisation & compliance

To ensure effective innovation in healthcare, a standardised legal process should be established. Notably, “to stimulate innovation in healthcare in a good way, it would be good to have a sort of standardised legal process that everybody’s using” (I11). In addition to that standardisation, regular audits play a crucial role in maintaining compliance. It was highlighted that “there will be some audits making sure that they are also compliant” (I8). Interviewee 11 reinforced the importance of accountability through established audit frameworks, explaining, “if you have an ISO 27001 or an N7510, or these kinds of things, you do regular audits to check on the accountability. I think that works, so that is a good way.” Implementing standardised legal processes and compliance audits can help create a more secure and efficient healthcare innovation ecosystem.

##### 4.2.1.3 Public engagement & awareness

Raising public awareness is essential to ensure that citizens understand how their data is collected and used. Interviewee 8 emphasises the importance of informing people about data collection and their rights, stating the importance of “raising awareness on a society level […] for citizens to know that this data is being collected [and that] there is an opt-out option.” Beyond awareness, maintaining trust and engagement requires ongoing communication with individuals whose data is being used. The need for transparency and long-term engagement, explaining, “if you really want to get patients on board for a longer term […] you always have to give them feedback. […] So this feedback - and this is also the sense of understanding what is happening with their data - [is] why they were altruistic or they showed solidarity” (I7). Without such efforts, data collection risks becoming meaningless to the individuals involved: “citizens should know that their data are being collected and used and reused […] Otherwise, I think [the EHDS] will be like an empty shell” (I8). These perspectives highlight the importance of raising awareness and fostering an ongoing relationship of trust through transparency and feedback.

##### 4.2.1.4 Consent & opt-out mechanisms

Ensuring transparency and user control over data sharing requires a structured and accessible approach to information and consent mechanisms. In response, it was suggested to “layer information” such that “on a website, [there is] some very basic information” and “if people are not interested at all, they can even skip it. And then you can fold out more information, and then [even] more information for those of us who are really very concerned about privacy” (I7). This method allows users to engage with privacy details at their preferred level of depth. Interviewee 7 also emphasises the importance of defining specific opt-in categories, particularly for genomic data, mental health data, and behavioural data, explaining that “these are by definition sensitive, extra sensitive.” The suggestion was made to make “opt-outs, opt-ins […] more mixed and more granular.”

However, the effectiveness of opt-in and opt-out mechanisms depends on their usability. Interviewee 8 raised concerns about the practicality of opt-out systems, questioning, “whether the opt-out is as easy to use as when you did not opt-out at the very beginning. So do you send reminders, or do people have to go with it? They forget about it.” To improve flexibility, Interviewee 12 proposes an adaptive opt-in/opt-out system that allows individuals to make decisions on a case-by-case basis, stating, “we are more looking at a different approach that you have a single switch opt-out, opt-in for all health data to be used for secondary use for research health and innovation. [Such that] for each attempt that your data is requested that you are able to opt out.”

A centralised administration for managing opt-in and opt-out requests could streamline the process and reduce the burden on individuals. Interviewee 12 highlights the advantages of such a system: “instead of making an opt-out or opt-in with each different health Institute, go to the centralised administration, [which] will give the data subject less administrative burden. Also will give this person the opportunity to have insight into what is happening with the data about them.” Finally, simplifying the process is crucial, as Interviewee 10 suggests, to “fit it all on one page,” reinforcing the need for clarity and ease of use in data-sharing decisions.

#### 4.2.2 Technological

##### 4.2.2.1 Access control & data minimisation

Controlled access and data minimisation are key measures to address data security concerns, such as inefficient authentication, privacy breaches, and the risks associated with secondary use of health data under the EHDS. Ensuring secure access begins with robust credential and access control mechanisms, as Interviewee 13 emphasises: “this starts with the right credential and that the person who gets access…is granted on a legal basis.” Similarly, Interviewee 14 highlights the need for an accreditation process to strengthen authentication, stating: “you have the whole identification and authentication process…so you know who is exactly applying for permits and accessing your data.” Effective identity and access management further requires clear permit criteria, defining “what can you do on which data, on what level, for how long, from which environment” (I14).

To prevent unauthorised dissemination, controlled environments ensure data remains secure and traceable. As Interviewee 14 asserts, “data should be accessible indirectly only…it should be in controlled environments at all times.” Additionally, the principle of data minimisation, keeping data as close to its source as possible, was emphasised by Interviewee 2: “you keep it at the sensor itself…keep it as much as possible at its source.”

In line with ensuring accountability and transparency, interviewees highlighted the importance of implementing systematic logging mechanisms. Logging serves not only as a technical tool but also as a means to uphold governance principles by recording who accesses data and under what conditions. As one interviewee put it, “you will always log your credentials when you access information…such a system could…help with accountability and logging” (I13). Another noted that logging could be a key measure to mitigate risks by “log[ging] every data request…in a system that belongs to the patient” (I10). Although seen primarily as a technical implementation, logging was cited as essential for fostering trust and ensuring compliance through traceable access records (I10; I13).

##### 4.2.2.2 Federated systems & privacy-enhancing technologies

Federated systems and Privacy-Enhancing Technologies (PETs) are highlighted as essential solutions to strengthen data protection while enabling meaningful analysis (I2; I3; I13; I16). Interviewee 2 stresses the importance of enforcing PETs. Specifically, using “advanced security technologies, such as homomorphic encryption, could enhance data protection while allowing for analysis. It limits the information that is made available for analysis.” Similarly, Interviewee 13 advocates for early integration of these tools, arguing that “[privacy-enhancing] technologies are very useful and [that] they actually should be implemented from drafting [the technical and governance architecture] already.”

Likewise, a federated approach was repeatedly emphasised as a way to enhance security while minimising direct data exposure. Interviewee 13 noted that such a system “should allow for more safety,” while Interviewee 3 points out its ethical advantages, explaining that “federated ways of sharing data…lead to fewer challenges on ethics purposes.” Federated learning, in particular, was highlighted as a way to prevent unnecessary data transfers. As Interviewee 3 explains, “if we use more federated learning, then you are not really releasing personal data to the researcher.” This principle extends to federated analysis environments, where research can be conducted without requiring centralised data storage. Interviewee 12 describes such an approach: “it could be a federated analysis environment. It doesn’t have to be a centralised solution. So a secure process environment can still be dealt with locally with a federated approach.” These measures aim to reduce unnecessary data exposure while maintaining usability for research and analysis.

##### 4.2.2.3 Data quality assurance

Ensuring data quality is another aspect of facilitating the secure and effective secondary use of health data within the EHDS. A key initiative in this regard is the “Quantum project” according to interviewees (I2; I12), which aims to establish a standardised quality label for data holders. As Interviewee 2 explains, Quantum seeks to define “a label regarding the quality [and] the obligation of the data holder,” a point also emphasised by Interviewee 12. By introducing standardised “quality assurance measures” (I11), such initiatives help enhance data reliability, ensuring that secondary use is based on accurate, well-maintained and trustworthy datasets (I2; I11; I12).

### 4.3 Privacy-enhancing technologies adoption

The interviews reveal significant gaps in the adoption of PETs within the EHDS, and many participants highlight the limited depth of knowledge and development. Several interviewees noted that PETs, such as federated learning, are still largely in theoretical stages (I5, I16), and the necessary expertise is not widespread (I11). This suggests a need for further research into knowledge dissemination and training frameworks to address these gaps.

A recurring theme is the absence of standardised frameworks for the implementation of PETs (I4, I14, I15), particularly in relation to data ownership and access. As one interviewee states, “there should be a clearer rule set about which data needs to be separate from other data” (I14). This implies that research into the development of regulatory frameworks and best practices for PETs would be highly valuable.

Concerns about the trade-off between security and user-friendliness also emerged, with one interviewee noting, “if something becomes more secure, it [often] also becomes less user-friendly” (I4). This need for more accessible PET usage for researchers is highlighted by other interviewees as well (I7, I8).

Finally, the need for automatic enforcement of PETs was emphasised. An interviewee remarked: “you shouldn’t be the one that applies this technology; you should use a system where these privacy-enhancing technologies are enforced” (I2). This indicates the need for research to create systems that automatically integrate PET, reducing the dependence on researchers for manual implementation.

## 5 Discussion

### 5.1 Reflection on the risk-solution map

**Figure 2:**
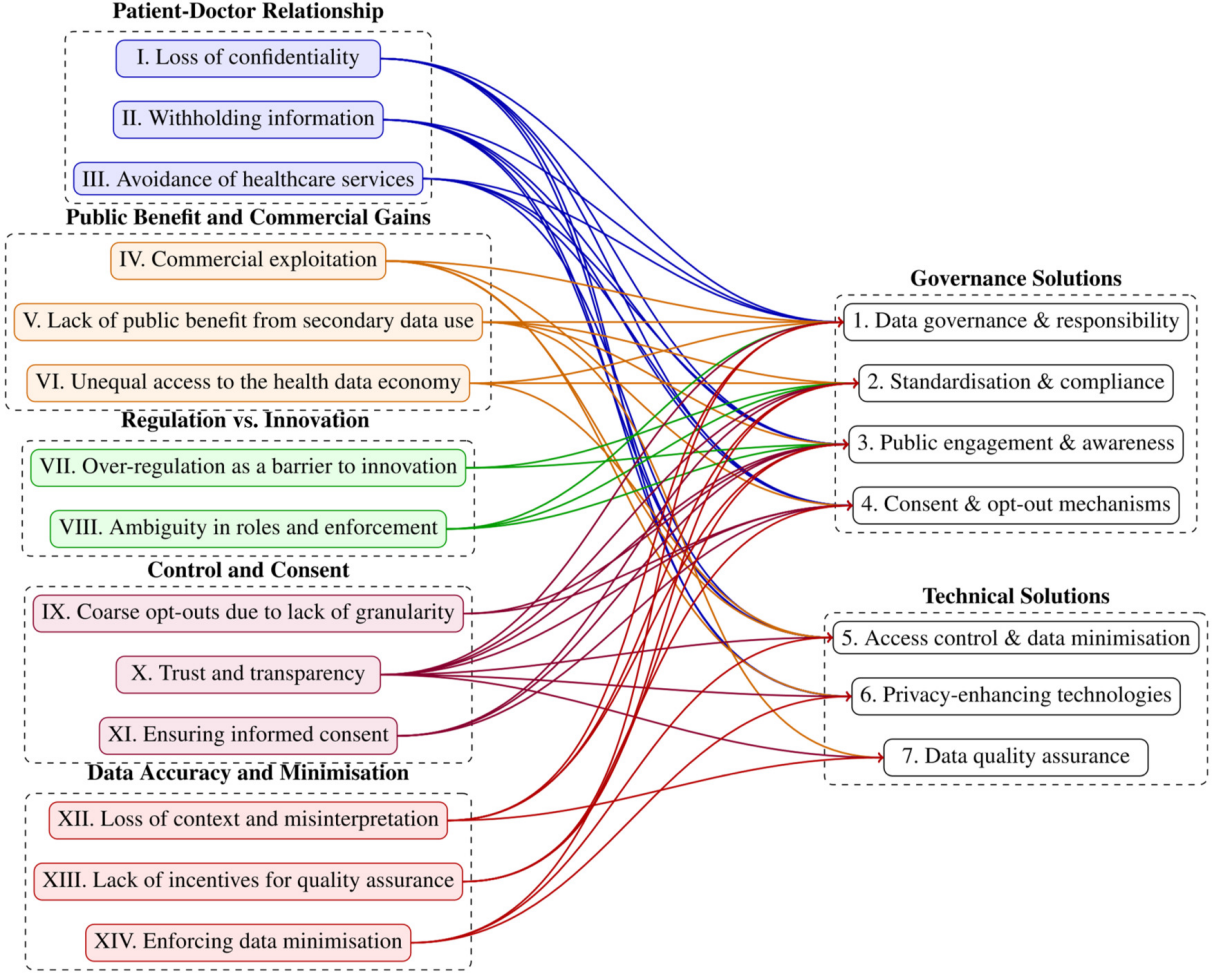
Color-Coded Mapping from Risks (Left) to Solutions (Right).

The interviews allowed us to map the solutions to risks. This mapping process was carried out on an iterative basis by the first four authors, each bringing their domain-specific expertise to the task. The researchers collaboratively linked solutions to corresponding risks, with the process grounded in their collective judgment and interpretation. The mapping exercise is presented in Figure 2. For a detailed overview of which specific risks and solutions are linked, a corresponding table is provided in the Supplementary Material, as not all relationships can be clearly traced in this figure.

The EHDS introduces a transformative regulatory framework for European health data governance, whose effectiveness depends on the calibration of risk management and data utility (5, 31, 32). Our study highlights five concerns, fourteen related risks and seven solution directions associated with its implementation, particularly in data governance, privacy, and the execution of data-sharing mechanisms for secondary use of health data as enshrined in the data permits. While we initially examined the challenges independently, a key finding is the interconnectedness of risks and solutions (refer to Figure 2). Our results reveal that several solutions link to multiple concerns. Most notably “public engagement and awareness”, which address eleven of the identified challenges, underscoring their central role in mitigating systemic risks. This strong interdependence highlights the necessity of an integrated strategy for EHDS implementation, prioritising highly correlated solutions that are likely to yield the greatest impact. We further elaborate on the identified risks, putting them within the broader context of existing literature.

The identified risks and solutions are consistent with existing literature, which underscores that large-scale health data initiatives thrive only when clear regulatory frameworks, trust, and robust technological safeguards are in place (31, 33, 34). However, our study adds a new dimension by emphasizing the structural imbalances in access to the health data economy, revealing the risk of reinforcing disparities rather than democratizing data access.

This highlights an important balance that is also likely to be relevant beyond the EHDS - i.e., the balance between over-regulation and effective regulation. On one hand, the EHDS raises concerns that it adds to the regulatory burden already faced, for instance, by hospitals or smaller companies. Our study highlights the operational burdens the EHDS places on healthcare providers. The administrative and technical requirements — ranging from interoperability standards to compliance costs — pose substantial challenges, particularly for smaller organizations with limited resources. This resonates with findings from digital health governance research, which warns that while policy-driven interoperability initiatives aim to streamline data exchange, they often overlook the real-world constraints of healthcare providers (5, 35). Our study reinforces this concern but expands on it by showing how smaller enterprises and non-dominant actors may struggle to participate in the health data economy, exacerbating existing inequalities.

On the other hand, the potential ambiguity of the legislation raises a separate set of concerns about how effective its definitions and frameworks shall be. One of the concerns raised in our analysis, for instance, is the ambiguity in key definitions – such as “data holder,” “electronic health data,” and “public benefit” – within the EHDS. This lack of clarity, also noted in recent regulatory critiques (8, 9), creates room for divergent interpretations across member states, ultimately hindering the uniform application of EHDS rules. Furthermore, the broad scope of the regulation — including both clinical and wellness-related data — raises significant privacy concerns.

While the EHDS builds upon the GDPR, our findings suggest that the current framework does not sufficiently address the unique challenges posed by secondary data use in health research. This aligns with ongoing debates on how to balance the societal benefits of secondary data use with individual rights and informed consent (8, 10), and highlights the risks associated with making additional legislation, especially in a regulatory landscape as complex as healthcare and use of secondary data.

Another issue that emerges is the potential erosion of patient-doctor confidentiality and its implications for trust in healthcare. Prior research has already highlighted that patients’ willingness to share data depends on perceived control and transparency (36). Our findings go further by demonstrating that the lack of a clear definition of “public benefit” in the EHDS exacerbates this distrust. Without transparent communication of how data will be used and for whose gain, public scepticism could undermine participation, reducing the effectiveness of the EHDS in fostering health data-driven research and innovation.

Addressing these risks requires solutions that are both governance- and technology-driven. On the governance side, clearer legal definitions and harmonized policies across member states are essential to prevent regulatory fragmentation. Additionally, a more explicit articulation of “public benefit”—grounded in democratic deliberation and citizen engagement— along with mandatory mechanisms or commitments to ensure such public benefit could help strengthen trust. On the technology side, PETs such as federated learning offer promising solutions to mitigate privacy risks while enabling secure data use [see Raab et al. (37)]. However, our findings reinforce that technology alone cannot replace strong governance. Instead, it should serve as a supportive mechanism for operationalising ethical and legal principles. Crucially, there is also an intersectional aspect between the governance- and technology-driven solutions, which is particularly relevant in, for instance, ensuring the understandability of and equal access to the legislation.

Finally, our study underscores the need to view data permits within a broader governance framework. Rather than treating them as isolated regulatory tools, data permits should be embedded within a comprehensive strategy for responsible data stewardship. This aligns with recent research advocates for participatory governance models (38), where transparency, stakeholder engagement, and continuous risk assessment guide the ethical use of health data (39).

### 5.2 A research agenda for the role of privacy-enhancing technologies within the EHDS

The interviewees stated that technological solutions, like Privacy-Enhancing Technologies (PETs), are an important solution to some of the concerns. This aligns with previous studies that emphasize the growing need for robust data privacy measures in healthcare settings, particularly with the increasing amount of sensitive health data being shared across various platforms (16).

For the EHDS in particular, we observe that PETs can play an important role at various stages, if implemented with appropriate clear governance and stakeholder engagement strategies. However, the knowledge on this subject remains limited, with a lack of comprehensive understanding regarding their practical application, integration, and scalability within the European health data framework. Further investigation is needed to determine how these technologies can be effectively implemented to balance both privacy and accessibility in the EHDS ecosystem. PETs, we argue, should be built directly into the governing apparatus of the EHDS, and be taken into account directly from the start in line with the privacy-by-design approach, ensuring that data protection is embedded into the system’s architecture and vice-versa, rather than added as an afterthought.

Furthermore, it is important to note that the concerns we have identified are interconnected - i.e., addressing them in isolation, and especially without a clear understanding of how technical and governance aspects interact - is likely ineffective and might even cause harm rather than good. For example, as important as it is to ensure that patient data is protected through the appropriate PETs (Concerns over data security, Concerns over the patient-doctor relationship), it is also equally important to identify ways to communicate this to the patient in a way that enables their trust (Concerns over consent & control). Similarly, detangling concerns related to public benefit vs. commercial gains also requires a balanced approach to concerns related to over- and ambiguous regulation.

In response, we propose an agenda for socio-technical research. This agenda aims to explore the technical, regulatory, and societal aspects of PETs in healthcare, focusing on developing actionable solutions that enhance privacy while enabling the secure sharing and use of health data across borders, also at the intersection of governance and technology. The agenda is the following:

#### 5.2.1 Privacy patterns within the EHDS

Privacy patterns offers a clear framework and guidelines for managing specific privacy issues such as data access, consent, and security. Such patterns are developed to ensure consistency in privacy practices, helping stakeholders—whether they are policymakers, developers, or organisations—communicate effectively and implement solutions in a systematic manner (40, 41). However, no studies so far how privacy patterns can support the processes within the EHDS.

For example, the “Data Minimisation” pattern advocates for collecting only the minimal amount of data necessary for a given purpose. This pattern helps mitigate the risks associated with over-collection and excessive sharing of personal data. It is an essential tool in the development of PETs, supporting data protection while ensuring compliance with privacy regulations like GDPR [see also Quinn et al. (8)].

Based on the results of our interviews, privacy patterns address several research gaps in the adoption of PETs within the EHDS. Key issues raised by interviewees include the limited knowledge and development of PETs (I5, I16, I11), the absence of standardized frameworks (I4, I14, I15), and the trade-off between security and user-friendliness (I4). Privacy patterns, by providing a structured approach to designing and implementing PETs, can fill these gaps by offering clear solutions for data governance, compliance, consent mechanisms, and access control.

For instance, the “Access Control” pattern offers guidelines on how to restrict access to sensitive health data, ensuring that only authorized individuals or systems can view or modify data. This aligns with the need for stronger regulatory frameworks for data ownership and access (I14) while supporting the development of secure, yet user-friendly, PETs. Furthermore, privacy patterns can contribute to automatic enforcement of PETs, addressing concerns about the manual application of privacy technologies (I2). Through these standardized solutions, privacy patterns can enhance the interoperability, usability, and adoption of PETs in health data management.

#### 5.2.2 Accessible rules, and rules as code

As highlighted in the interviews, gaps exist in level of understanding different stakeholders have about the EHDS in general and PETs in specific, including absence of standardised frameworks for data ownership and access (I4, I14). Different resources can and should be developed to make the process of navigating legal systems easier for stakeholders. Simple, low-tech resources (such as the the Finish Social and Health Data Permit Authority website https://findata.fi/en/) can be developed to address these gaps by providing clear, accessible guidance on rights and responsibilities, simplifying complex governance rules, and helping different actors navigate data governance and compliance, particularly for small and medium enterprises (SMEs) and patients. Furthermore, research from the field of legal informatics or “rules as code” (42) presents a promising line of inquiry with regards to harmonising regulatory frameworks and enhance stakeholder engagement. Research in this field aligns closely with the spirit of the privacy-by-design process, and can assist in building PETs directly into governance structures.

#### 5.2.3 Integrating PETs into the data permit process

To simplify governance in the EHDS and ensure the smooth flow of data, we propose integrating PET into the Data Permit process. This approach directly embeds privacy protections into the data-sharing architecture, ensuring that privacy is considered throughout the entire data flow, rather than as an afterthought. Integrating PETs into the Data Permit process can provide clear, enforceable agreements on data access, usage, and consent, reducing ambiguity and enhancing transparency. This would address the challenges raised in the interviews, particularly around the need for standardised frameworks for data ownership and access (I4, I14), as well as concerns regarding the trade-off between security and user-friendliness (I4).

By directly linking Data Permits with technical privacy-enhancing measures, such as federated learning and access controls, the governance of health data can be both more secure and more efficient. The usage of PETs have been discussed in the past, though no clear execution rules or standards are proposed yet (12). This integrated approach not only aligns with the principles of privacy-by-design but also supports the automation of PETs, ensuring their consistent and reliable enforcement without burdening stakeholders, particularly SMEs and smaller healthcare providers, with additional administrative overhead.

In addition to access control, PETs such as Federated Learning and Multi-Party Computation can also serve as privacy-preserving pre-processing tools to assess the completeness and contextual richness of data prior to its use. This allows for an initial check of whether the available data is suitable for purpose, for instance, in terms of demographic coverage, time interval, or data modalities, without exposing sensitive content (43). These assessments could feed into the Data Permit process itself, informing both the granting decision and the definition of analysis conditions. This integration would strengthen the alignment between legal permissions and the actual utility and limitations of the data, thereby supporting more informed and proportionate data use decisions.

#### 5.2.4 Verification of models with zero-knowledge proofs

To address the critical issue of data accuracy in the EHDS, we propose the integration of zero-knowledge proofs (ZKPs) for the verification of AI models. ZKPs can be used to check whether an large langugage model (LLM) is making the right predictions—without actually showing how the model works or revealing any of its secret data (44). This method allows stakeholders to confirm the integrity and correctness of AI decisions without disclosing any confidential information, thereby ensuring privacy while maintaining trust in AI-driven health data systems. For instance, a healthcare provider could use ZKPs to prove that an AI model has correctly processed patient data and produced accurate results without exposing the actual data to external auditors. This approach aligns with existing research on enhancing privacy in AI systems, particularly in healthcare settings where data confidentiality is key (45). By implementing ZKPs, the EHDS can ensure that AI models remain transparent and trustworthy, while safeguarding the privacy of sensitive health data, thereby promoting the adoption of AI technologies in healthcare while addressing regulatory concerns over data misuse and accuracy.

## 6 Conclusion

This research presented an inductive, empirical approach rooted in semi-structured interviews to study the conditions for ethical secondary use of data under EHDS. The interviews revealed a number of concerns and risks associated with the EHDS. They also revealed potential solutions to these risks, on the level of governance and policy as well as technology.

The contributions of this research are two fold. First, it presents a mapping of said concerns and risks to prospective solutions (RQ1 and RQ2). This reveals the interconnectedness of risks and solutions, underscoring the central role of certain solutions, such as public engagement and awareness, in addressing these challenges. Additionally, the study introduces a new dimension to the concerns, focusing on the structural imbalances in access to the health data economy. Second, and in connection present literature, it provides a deep dive in the role played by PETs (RQ3). The second contribution comes in the form of a research agenda on PETs in the EHDS. We foresee future work to build on this exploratory research and engage with the research agenda.

As the EHDS is rolled out, numerous stakeholders in the health space will seek to address the concerns and risks posed. Within that context, this study acts as a compass - with emphasis on PETs - for the successful roll out of the data space.

### 6.1 Limitations

There are several limitations to this study that should be considered when interpreting the findings. Four of these are noteworthy.

First, the interviews were conducted before the finalisation of the EHDS. As a result, the participants’ insights may not fully reflect the most recent developments or final regulatory frameworks surrounding the EHDS. This could have influenced the relevance and applicability of their perspectives in the current context. We addressed this limitation by considering the maturity of the regulation as of February 2024.

Second, the sample size of interviewees was limited, which may impact the generalizability of the results (46). A larger, more diverse sample could provide a broader range of viewpoints and contribute to a more comprehensive understanding of the topic. We addressed this limitation by seeking a variety in the interviewees (including interviewees from healthcare institutions, ministries and academia).

Third, the data coding process may have been influenced by confirmation bias, where the researchers inadvertently focused on information that supported pre-existing hypotheses or expectations. To mitigate this limitation, we involved a fourth researcher in reviewing our initial draft findings, ensuring a critical evaluation of our results.

Fourth, most participants were based in the Netherlands, a country with a relatively strict regulatory environment for health data reuse (47). Although this may limit generalisability to more permissive contexts, it offers valuable insight into challenges in stricter regimes. Many of the identified issues, such as balancing privacy with public benefit, are likely to resonate in different governance settings, though with varying emphasis.

## Data Availability

The anonymised raw data supporting the conclusions of this article will be made available by the authors upon reasonable request.

## Footnotes

^1^It is important to note here that “anonymised” is a legally significant term, and that the EHDS suggests to primarily use anonymised data, with psuedonymous data being used only when this is not possible. There are, currently, two perspectives on the anonymisation of data under the GDPR: (1) that personal data, including health data, is unlikely to be rendered completely anonymous in the strict sense defined by the GDPR in its Recital 26 (“*information which does not relate to an identified or identifiable natural person or to personal data rendered anonymous in such a manner that the data subject is not or no longer identifiable*”), and (2) a more recent and nuanced interpretation, which accepts some risk of re-identification of the data, if such re-identification is still not likely or reasonable. The EHDS *may* be leaning towards the latter interpretation, in particular in its phrasing of Recitals 72 and 92, and because the EHDS foresees that data correction, annotation, and enrichment should be transmitted to the original data holder (EHDS, Recital 57), which would only be useful if a level of traceability was retained. We clarify that it is not the goal of this article to explore whether the data for secondary use under the EHDS is irrefutably anonymous. We assume a harmonious interpretation between the EHDS and the GDPR.
