# Supplementary material for: Secondary use under the European Health Data Space: setting the scene and towards a research agenda on privacy-enhancing technologies
Source: Front Digit Health. 2025 Jun 19;7:1602101. doi: 10.3389/fdgth.2025.1602101 (PMC12222193; doi:10.3389/fdgth.2025.1602101)
Supplement: Supplementary file 1 [file Datasheet1.pdf]

Supplementary Material

Interview	Affiliation
Interviewee 1	Non profit service provider (Infrastructure Architect, FIN)
Interviewee 2	Academia (Health Data Strategist, ES)
Interviewee 3	Hospital (Scientific coordinator, NL)
Interviewee 4	Non profit (Public Health Researcher, NL)
Interviewee 5	Academia (Data scientist, NL)
Interviewee 6	Hospital (Medical doctor, NL)
Interviewee 7	Patient advocacy group (Digital Health Advocate, NL)
Interviewee 8	Hospital (Postdoc Bioethics and Law, ES/NL)
Interviewee 9	Academia (Associate Professor Law, BE/NL)
Interviewee 10	Patient advocacy group (Policy Advisor, NL)
Interviewee 11	Industry (Commercial Sensor Provider, NL)
Interviewee 12	Non profit service provider (Infrastructure Architect, NL)
Interviewee 13	Standardisation body (Digital Health Advisor, NL)
Interviewee 14	Hospital (Infrastructure Architect, NL)
Interviewee 15	Hospital (DPO, NL)
Interviewee 16	HDAB (Project lead, NL)

Table 1: Interviewees (anonymised) and their affiliations.

1.1 Interviews.

1.2 Question guide. 1. Data Processing

- In your current practice, what would a typical data flow for the Box-plus look like?
- What steps are usually taken in order to make data available to other parties?
- How is data collected from different sources (internally and externally)?

2. Opportunities for Reuse

- What are the potential benefits for each stakeholder (e.g., data holder, data requester, health practitioner, patient, infrastructure provider) in this scenario if Box-plus data is reused for secondary purposes (research, innovation, policy)?
- Do the opportunities for each stakeholder differ? Do they conflict?

3. Risks of Reuse

- What risks do you see for each stakeholder (e.g., data holder, data requester, health practitioner, patient, infrastructure provider) if Box-plus data is reused for secondary purposes?
- Do the risks for each stakeholder differ?

- How significant are these risks according to you?

4. **Criteria for Data Permit Evaluation**

- Based on the opportunities and risks, what criteria should the Data Permit request further information about from the Data Requester to balance secondary use with respect to privacy and societal norms?
  - Are any or some of these criteria often overlooked?
  - Which of these criteria is or are most important?
  - Are there any that are less important or not important?

5. **GDPR Criteria Evaluation**

- Reviewing the key principles of the GDPR, would you add or remove any criteria for the EHDS Data Permit process, and why?
  - Lawfulness, Fairness, Transparency
  - Purpose Limitation
  - Data Minimization
  - Accuracy
  - Storage Limitation
  - Integrity and Confidentiality
  - Accountability

6. **Questions about technologies**

- To what extent should tools and techniques be included as criteria in the Data Permit application?
- Are privacy-enhancing technologies appropriate, and why/why not?

1.3 Linkage risks and solutions.

Risk / Solution	1 Data Governance & Responsibility	2 Standardization & Compliance	3 Public Engagement & Awareness	4 Consent & Opt-out Mechanisms	5 Access Control & Data Minimisation	6 Federated Systems & Privacy-enhancing Technologies	7 Data Quality Assurance
**Patient-doctor relationship**							
I Loss of confidentiality	✓		✓	✓	✓	✓	
II Withholding information	✓		✓	✓	✓	✓	
III Avoidance of healthcare services	✓		✓	✓	✓		
**Public benefit and commercial gains**							
IV Commercial exploitation	✓			✓	✓	✓	
V Lack of public benefit from data reuse	✓	✓	✓				✓
VI Unequal access to the health data economy	✓	✓			✓		
**Regulation versus innovative capacity**							
VI Over-regulation as a barrier to innovation		✓	✓				
VIII Ambiguity in roles, responsibilities	✓	✓	✓				
**Control and consent**							
IX Coarse opt-outs due to lack of granularity			✓	✓			
X Trust and transparency	✓	✓	✓	✓	✓	✓	✓
XI Ensuring informed consent		✓	✓	✓			
**Data accuracy and minimisation**							
XII Loss of context and misinterpretation	✓	✓	✓				✓
XIII Lack of incentives for quality assurance	✓	✓	✓				
XIV Enforcing data minimisation				✓	✓	✓	

Table 2: Mapping Risks to Solutions
